# Integration of Biomechanical Analysis and Motion Capture Technology in Physical Therapy and Rehabilitation: A Systematic Review

**DOI:** 10.7759/cureus.115426

**Published:** 2026-08-30

**Authors:** Chetana A Pawar, Sandeep Shinde, Durga A Jagdale

**Affiliations:** 1 Department of Musculoskeletal Sciences, Krishna College of Physiotherapy, Krishna Vishwa Vidyapeeth (Deemed to Be University), Karad, IND

**Keywords:** biomechanical analysis, biomechanics, gait analysis, markerless motion capture, motion capture, physical therapy, virtual reality, wearable sensors

## Abstract

Biomechanical analysis and motion-capture technology have emerged as valuable tools for the objective assessment of human movement in physical therapy and rehabilitation. These technologies provide quantitative information on gait, balance, posture, joint kinematics, and functional performance, thereby supporting clinical decision-making and individualized rehabilitation. Despite increasing clinical adoption, evidence regarding their effectiveness across different rehabilitation populations remains heterogeneous. This systematic review aimed to evaluate the integration of biomechanical analysis and motion-capture technology in physical therapy and rehabilitation. A systematic literature search was conducted across major electronic databases to identify randomized controlled trials evaluating the integration of biomechanical analysis and motion-capture technologies within physical therapy and rehabilitation. Studies that investigated biomechanical analysis, motion-capture systems, wearable sensors, virtual reality, and markerless motion-capture technologies in physical therapy interventions were considered. Methodological quality was assessed using the Cochrane Risk of Bias 2 tool, and a narrative synthesis was performed because of clinical and methodological heterogeneity. The evidence encompassed neurological, musculoskeletal, pediatric, geriatric, and oncological rehabilitation populations. Interventions incorporated Kinect-based rehabilitation, markerless motion capture, wearable sensor systems, virtual reality, robot-assisted gait training, movement biofeedback, and sensor-augmented telerehabilitation. Overall, the evidence indicated improvements in gait, balance, postural control, movement quality, functional mobility, limb-loading symmetry, upper limb function, and quality of life across various rehabilitation settings. Risk of bias assessment indicated generally low risk of bias or some concerns, although concerns related to deviations from intended interventions and reporting practices were observed across the evidence base. The integration of biomechanical analysis and motion-capture technology may enhance objective movement assessment and support functional recovery across diverse rehabilitation populations. These technologies may facilitate individualized rehabilitation and evidence-based clinical decision-making through quantitative movement analysis and feedback. However, methodological heterogeneity highlights the need for standardized assessment protocols and high-quality trials to establish their long-term clinical effectiveness and support broader implementation in rehabilitation practice.

## Introduction and background

Physical therapy and rehabilitation aim to restore movement, improve functional independence, minimize disability, and enhance the quality of life of individuals with musculoskeletal, neurological, cardiopulmonary, and sports-related disorders. Accurate assessment of human movement is fundamental to effective rehabilitation, as it facilitates the identification of movement impairments, supports individualized treatment planning, enables recovery monitoring, and assists in evaluating treatment outcomes. Traditionally, movement assessment has relied on observational gait analysis and standardized clinical outcome measures. Although these approaches are widely used for their simplicity and low cost, they are inherently subjective and may be influenced by examiners' experience, leading to interrater variability and limited sensitivity in detecting subtle biomechanical abnormalities. Consequently, there has been increasing interest in incorporating objective biomechanical assessment technologies into routine rehabilitation practice to improve the accuracy, reliability, and reproducibility of clinical evaluations [1].

Motion capture technology has become an essential component of biomechanical analysis in rehabilitation. Conventional marker-based optical motion capture systems remain the reference standard for three-dimensional movement analysis due to their high accuracy. However, their clinical application is limited by the need for specialized laboratory facilities, multiple infrared cameras, reflective markers, sophisticated software, and trained personnel. These requirements increase operational costs and restrict their routine implementation in many rehabilitation settings. As a result, considerable research has focused on developing low-cost, portable, and clinically feasible alternatives without compromising measurement accuracy [1].

Recent technological advances in computer vision, artificial intelligence (AI), machine learning, wearable sensors, inertial measurement units (IMUs), depth-sensing cameras, and markerless motion capture systems have transformed the assessment of human movement. Unlike conventional systems, markerless motion capture technologies estimate body movement directly from video images or depth sensors without the use of reflective markers. These systems reduce patient preparation time, improve patient comfort, simplify data collection, and enable movement assessment in hospitals, outpatient clinics, community settings, and home-based rehabilitation environments. A recent systematic review reported that markerless motion capture technology demonstrates acceptable validity and reliability in evaluating gait, balance, posture, upper extremity function, sit-to-stand performance, and other clinically relevant movement parameters across diverse rehabilitation populations [2].

Biomechanical analysis involves the quantitative evaluation of human movement by assessing kinematic, kinetic, temporal-spatial, and neuromuscular parameters. It provides objective information regarding joint motion, muscle performance, gait characteristics, postural control, balance, and movement symmetry. Such quantitative data assist clinicians in identifying abnormal movement patterns, understanding the mechanisms underlying functional impairments, monitoring patient progress, and evaluating the effectiveness of rehabilitation interventions. The integration of biomechanical analysis into physical therapy has strengthened evidence-based clinical practice by providing objective measures that complement conventional clinical assessments [1,3]. The evolution of movement assessment from traditional clinical methods to advanced biomechanical analysis and motion-capture technologies is illustrated in Figure 1.

**Figure 1:**
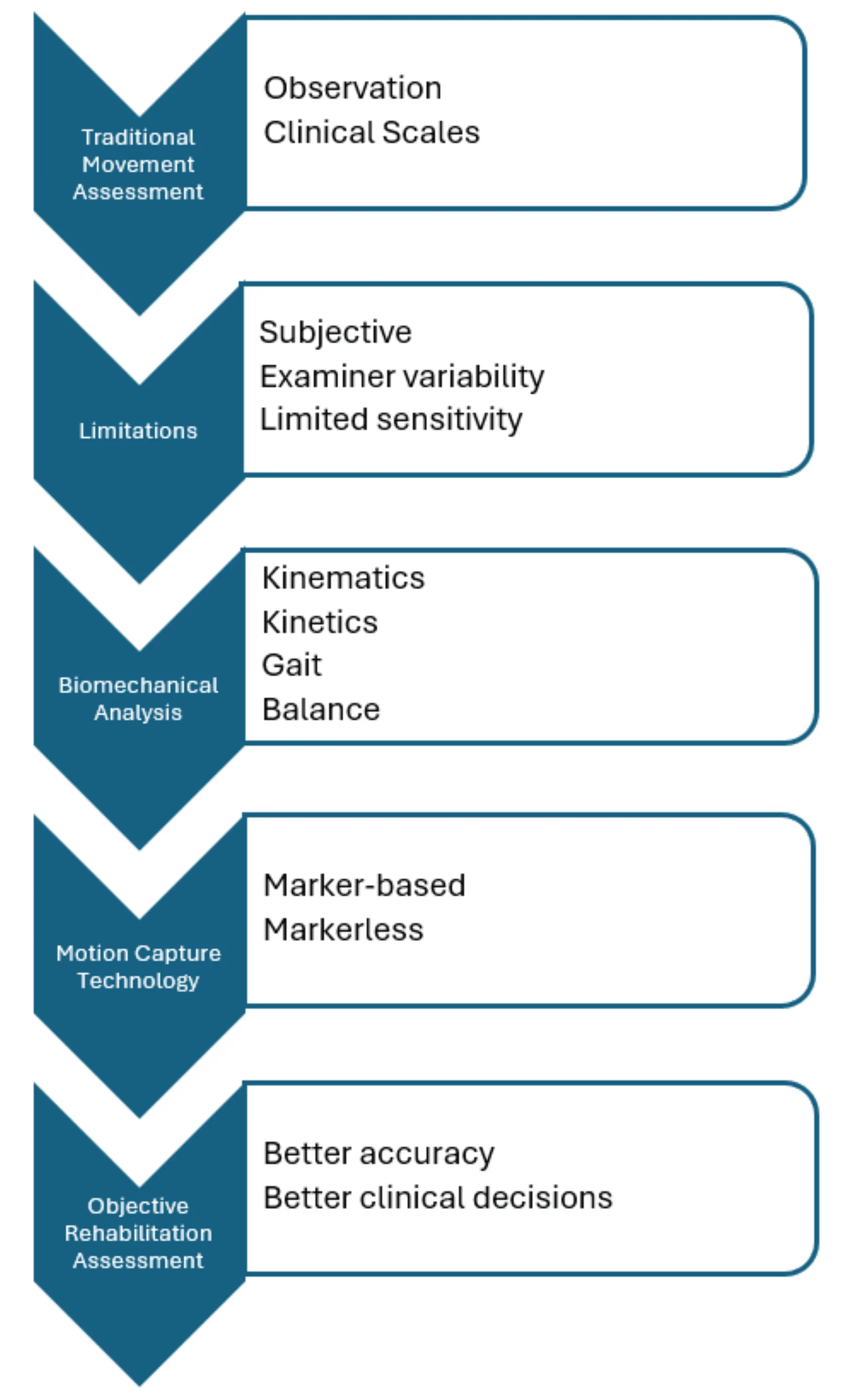
Evolution from traditional movement assessment to biomechanical analysis and motion capture technology in physical therapy and rehabilitation Image credit: This image was created by Chetana A. Pawar using Microsoft PowerPoint (Microsoft Corporation, Redmond, WA)

The integration of computational approaches has further expanded the capabilities of modern rehabilitation technologies. Machine learning algorithms, computer vision techniques, and AI enable automated recognition and evaluation of rehabilitation exercises, objective assessment of movement quality, real-time performance feedback, and remote monitoring of patient adherence. These technologies facilitate personalized rehabilitation programs, improve clinical decision-making, and support the growing demand for telerehabilitation and home-based rehabilitation services. Liao et al. highlighted the increasing role of computational approaches in providing objective, efficient, and automated assessment of rehabilitation exercises [3].

Significant progress has also been achieved in developing biomechanical analysis pipelines for markerless motion capture systems. Recent studies have incorporated advanced pose estimation algorithms, trajectory optimization techniques, inverse kinematic modeling, and musculoskeletal simulations to improve the accuracy and reliability of markerless movement analysis. These methodological advances have enabled markerless systems to generate biomechanical data that approach the accuracy of conventional laboratory-based motion analysis while substantially reducing equipment costs and operational complexity [4,5].

AI has emerged as a key driver in the evolution of markerless motion capture technology. Deep learning-based pose estimation algorithms can automatically identify anatomical landmarks, reconstruct three-dimensional body movements, estimate joint kinematics, and quantify functional motor tasks using conventional digital cameras. Recent evidence suggests that AI-based markerless motion capture systems offer a practical, accessible, and cost-effective approach for objective movement assessment and have considerable potential for widespread implementation in rehabilitation practice [6].

Motion capture technology has demonstrated broad clinical applicability across neurological, musculoskeletal, pediatric, geriatric, and sports rehabilitation. In neurological rehabilitation, markerless motion capture has been successfully used to assess gait abnormalities, balance deficits, upper limb motor function, and functional mobility in individuals with stroke, Parkinson's disease, cerebral palsy, and other neurological disorders. Furthermore, emerging evidence indicates that these technologies can provide objective digital biomarkers for monitoring disease progression and evaluating rehabilitation outcomes in neurodegenerative disorders. A recent systematic review concluded that markerless motion capture offers valuable quantitative measures of functional performance and may improve clinical assessment and longitudinal monitoring in individuals with neurodegenerative diseases [7]. The major components and clinical applications of motion-capture technology across rehabilitation settings are illustrated in Figure 2.

**Figure 2:**
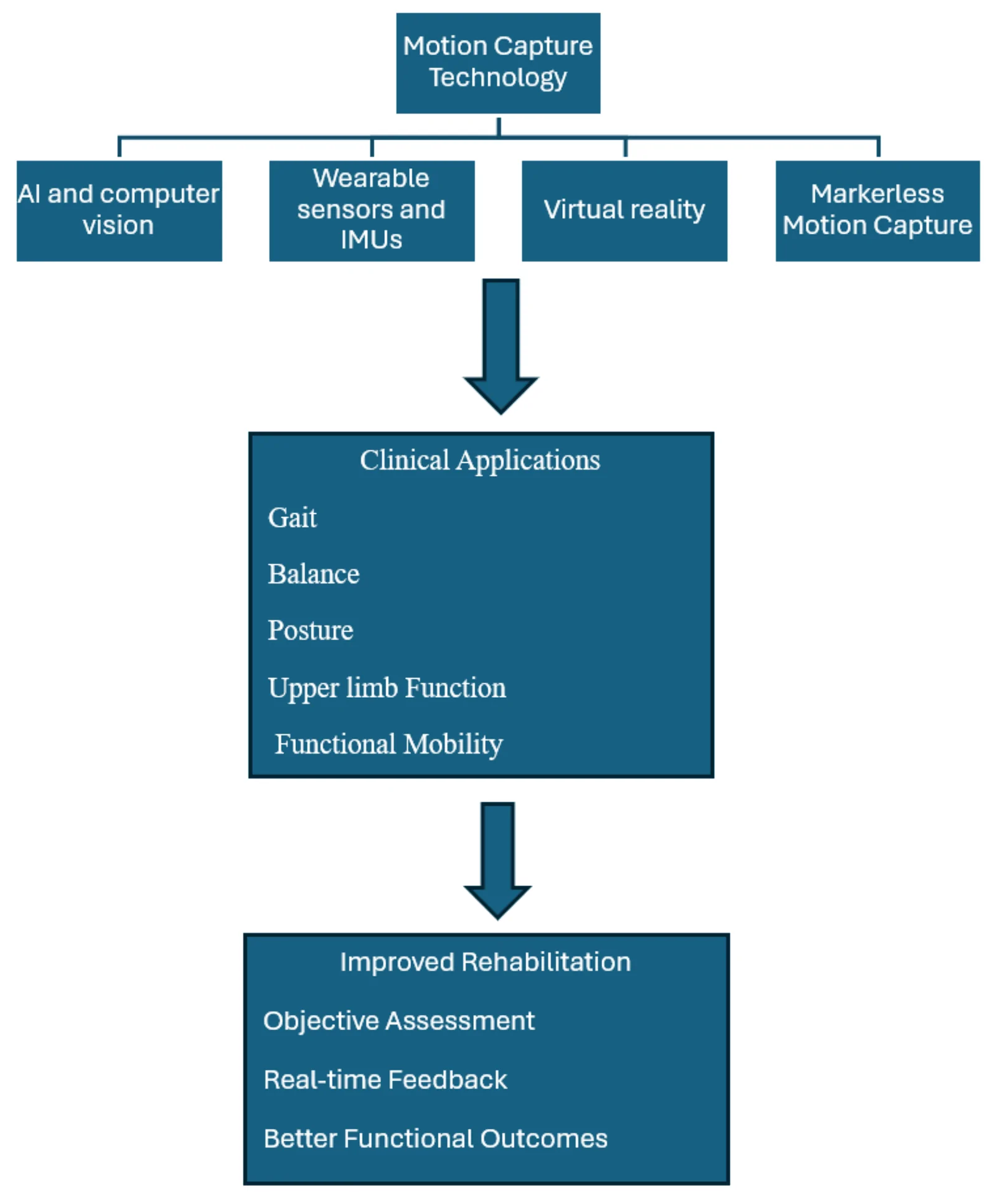
Components and clinical applications of motion capture technology AI: artificial intelligence; IMUs: inertial measurement units Image credit: This image was created by the author Chetana A. Pawar using Microsoft PowerPoint (Microsoft Corporation, Redmond, WA)

Despite these advancements, several challenges continue to limit the widespread clinical implementation of biomechanical analysis and motion capture technology. Variability in camera positioning, lighting conditions, calibration procedures, software algorithms, and outcome measures may influence measurement accuracy and reduce comparability across studies. Furthermore, methodological heterogeneity among published studies and the absence of standardized clinical protocols remain important barriers to routine implementation in rehabilitation practice [2,6]. Given the rapid technological evolution and expanding clinical applications of biomechanical analysis and motion capture systems, a comprehensive synthesis of the available evidence is warranted. Therefore, this systematic review aims to evaluate the integration of biomechanical analysis and motion capture technology in physical therapy and rehabilitation by summarizing current evidence regarding their clinical applications, effectiveness, advantages, limitations, and future directions. The findings of this review are expected to provide clinicians, researchers, and rehabilitation professionals with an evidence-based overview of the current role of objective movement analysis in contemporary rehabilitation practice and to identify areas requiring further research.

## Review

Methodology

Study Design

This systematic review was conducted and reported in accordance with the Preferred Reporting Items for Systematic Reviews and Meta-Analyses (PRISMA) 2020 guidelines [8].

Search Strategy

A comprehensive literature search was conducted to identify randomized controlled trials (RCTs) evaluating the integration of biomechanical analysis and motion-capture technology in physical therapy and rehabilitation. Electronic databases, including PubMed/MEDLINE, Scopus, Web of Science, PEDro, the Cochrane Central Register of Controlled Trials, and Google Scholar, were systematically searched from database inception through June 2026.

The search strategy combined controlled vocabulary, including Medical Subject Headings where applicable, with free-text keywords related to biomechanical analysis, motion-capture technology, and physical therapy or rehabilitation, using Boolean operators (AND and OR). The complete database-specific search strategies, including search terms, Boolean operators, filters, and search dates, are provided in Table 1. The PubMed/MEDLINE search strategy included: ("Biomechanical Analysis" OR biomechanics OR "movement analysis" OR "gait analysis" OR kinematics OR kinetics) AND ("Motion Capture" OR "Markerless Motion Capture" OR "Marker-Based Motion Capture" OR "Motion Analysis System" OR "3D Motion Analysis" OR Kinect OR "Leap Motion" OR "Wearable Sensor" OR "Inertial Measurement Unit" OR IMU OR "Virtual Reality" OR "Artificial Intelligence" OR "Computer Vision") AND ("Physical Therapy" OR Physiotherapy OR Rehabilitation OR "Exercise Therapy" OR "Neurological Rehabilitation" OR "Musculoskeletal Rehabilitation" OR "Sports Rehabilitation") AND ("Randomized Controlled Trial" OR randomized OR randomised OR RCT).

**Table 1:** Database-specific electronic search strategies CENTRAL: Cochrane Central Register of Controlled Trials; IMU: inertial measurement unit; PEDro: Physiotherapy Evidence Database; RCT: randomized controlled trial; TS: topic search; TITLE-ABS-KEY: title, abstract, and keywords

Database	Search strategy	Filters/limits applied
PubMed/MEDLINE	("Biomechanical Analysis" OR biomechanics OR "movement analysis" OR "gait analysis" OR kinematics OR kinetics) AND ("Motion Capture" OR "Markerless Motion Capture" OR "Marker-Based Motion Capture" OR "Motion Analysis System" OR "3D Motion Analysis" OR Kinect OR "Leap Motion" OR "Wearable Sensor" OR "Inertial Measurement Unit" OR IMU OR "Virtual Reality" OR "Artificial Intelligence" OR "Computer Vision") AND ("Physical Therapy" OR Physiotherapy OR Rehabilitation OR "Exercise Therapy" OR "Neurological Rehabilitation" OR "Musculoskeletal Rehabilitation" OR "Sports Rehabilitation") AND ("Randomized Controlled Trial" OR randomized OR randomised OR RCT)	English; Humans; database inception-June 2026
Scopus	TITLE-ABS-KEY (("biomechanical analysis" OR biomechanics OR "movement analysis" OR "gait analysis" OR kinematics OR kinetics) AND ("motion capture" OR "markerless motion capture" OR "marker-based motion capture" OR "motion analysis system" OR "3D motion analysis" OR Kinect OR "Leap Motion" OR "wearable sensor" OR "inertial measurement unit" OR IMU OR "virtual reality" OR "artificial intelligence" OR "computer vision") AND ("physical therapy" OR physiotherapy OR rehabilitation OR "exercise therapy" OR "neurological rehabilitation" OR "musculoskeletal rehabilitation" OR "sports rehabilitation") AND ("randomized controlled trial" OR randomized OR randomised OR RCT))	English; database inception-June 2026
Web of Science	TS = (("biomechanical analysis" OR biomechanics OR "movement analysis" OR "gait analysis" OR kinematics OR kinetics) AND ("motion capture" OR "markerless motion capture" OR "marker-based motion capture" OR "motion analysis system" OR "3D motion analysis" OR Kinect OR "Leap Motion" OR "wearable sensor" OR "inertial measurement unit" OR IMU OR "virtual reality" OR "artificial intelligence" OR "computer vision") AND ("physical therapy" OR physiotherapy OR rehabilitation OR "exercise therapy" OR "neurological rehabilitation" OR "musculoskeletal rehabilitation" OR "sports rehabilitation") AND ("randomized controlled trial" OR randomized OR randomised OR RCT))	English; database inception-June 2026
PEDro	(biomechanical analysis OR biomechanics OR movement analysis OR gait analysis OR motion capture OR markerless motion capture OR Kinect OR wearable sensor OR inertial measurement unit OR virtual reality OR artificial intelligence) AND (physiotherapy OR physical therapy OR rehabilitation)	Clinical Trial; English; database inception-June 2026
Cochrane CENTRAL	("biomechanical analysis" OR biomechanics OR "movement analysis" OR "gait analysis" OR kinematics OR kinetics) AND ("motion capture" OR "markerless motion capture" OR "marker-based motion capture" OR Kinect OR "Leap Motion" OR "wearable sensor" OR "inertial measurement unit" OR "virtual reality" OR "artificial intelligence" OR "computer vision") AND (physiotherapy OR "physical therapy" OR rehabilitation) AND ("randomized controlled trial" OR randomized OR randomised OR RCT)	English; database inception-June 2026
Google Scholar	("biomechanical analysis" OR "motion capture" OR "markerless motion capture" OR Kinect OR "wearable sensors" OR "virtual reality") AND (physiotherapy OR "physical therapy" OR rehabilitation) AND "randomized controlled trial"	English; database inception-June 2026

The search strategy was adapted to the indexing system and search syntax of each database while maintaining the core concepts of the search. In addition to electronic database searching, the reference lists of all included studies and relevant systematic reviews were manually screened to identify potentially eligible studies that were not retrieved through the database searches.

Studies were eligible if they were published in English, involved human participants, and were RCTs evaluating biomechanical analysis or motion-capture technologies within physical therapy or rehabilitation. Conference abstracts without full-text publications, editorials, letters to the editor, case reports, study protocols, narrative reviews, systematic reviews, meta-analyses, dissertations, and animal studies were excluded.

Grey literature sources and clinical trial registries, including the World Health Organization International Clinical Trials Registry Platform, were not searched because the review was restricted to peer-reviewed RCTs published in indexed electronic databases.

Study Selection and Data Extraction

All records identified through the electronic database search were imported into reference management software, and duplicate records were removed before screening. Two independent reviewers screened the titles and abstracts of the retrieved studies according to the predefined eligibility criteria. Full-text articles of potentially eligible studies were then assessed independently for inclusion. Any disagreements regarding study eligibility were resolved through discussion and consensus between the reviewers. Data were extracted independently by the same two reviewers using a standardized Microsoft Excel data extraction form. The extracted information included the first author's name, year of publication, study design, participant characteristics, sample size, diagnostic criteria, intervention details, comparator interventions, outcome measures, duration of follow-up, and main findings. Any discrepancies in data extraction were resolved through discussion to ensure accuracy and consistency.

Inclusion Criteria

Studies were included if they were RCTs published in peer-reviewed journals and written in English. Eligible studies involved human participants of any age or sex undergoing physical therapy or rehabilitation for musculoskeletal, neurological, sports-related, pediatric, geriatric, or other rehabilitation conditions. The intervention had to incorporate biomechanical analysis and/or motion capture technology, including marker-based or markerless motion capture systems, three-dimensional motion analysis, Kinect, Leap Motion Controller, wearable sensors, IMUs, virtual reality systems with motion tracking, or other technology-based movement analysis systems. Studies were required to report at least one rehabilitation-related outcome, such as gait parameters, balance, functional mobility, joint kinematics, movement quality, postural control, upper or lower limb function, pain, or quality of life. The inclusion and exclusion criteria used to determine study eligibility are presented in Table 2.

**Table 2:** Eligibility criteria for study selection VR: virtual reality; MR: mixed reality; IMU: inertial measurement unit

Criterion	Inclusion criteria	Exclusion criteria
Study design	Randomized controlled trials (parallel-group, single-blind, double-blind, multicenter, pilot randomized controlled trials)	Nonrandomized studies, cohort studies, case-control studies, cross-sectional studies, case reports, case series, narrative reviews, systematic reviews, meta-analyses, study protocols, conference abstracts
Population	Human participants of any age or sex undergoing physical therapy or rehabilitation for musculoskeletal, neurological, sports, pediatric, geriatric, or other rehabilitation conditions	Animal or cadaveric studies, healthy participants without a rehabilitation intervention, purely engineering or technical validation studies
Intervention	Rehabilitation interventions incorporating biomechanical analysis and/or motion capture technology, including marker-based or markerless motion capture, Kinect, Leap Motion, wearable sensors, IMUs, virtual reality, mixed reality, robot-assisted systems, mobile health, or sensor-based rehabilitation	Studies evaluating motion capture solely for diagnosis, surgical planning, ergonomics, sports performance, or technical validation without a rehabilitation intervention
Comparison	Conventional physiotherapy, usual care, sham intervention, no intervention, or other rehabilitation interventions	Studies without a comparator group or with comparisons unrelated to rehabilitation
Outcomes	Biomechanical and rehabilitation outcomes including gait parameters, joint kinematics, balance, postural control, functional mobility, movement quality, limb function, pain, quality of life, or rehabilitation performance	Studies reporting only technical performance (e.g., algorithm accuracy, camera calibration) without clinical rehabilitation outcomes
Language and setting	Full-text articles published in English in peer-reviewed journals; clinical, rehabilitation, outpatient, hospital, or community settings	Non-English publications, conference abstracts without full text, editorials, letters, dissertations, and animal or laboratory-only studies

Exclusion Criteria

Studies were excluded if they were non-randomized studies, observational studies, cohort studies, case-control studies, case reports, case series, conference abstracts without full-text publications, study protocols, editorials, letters, narrative reviews, systematic reviews, or meta-analyses. Studies involving animal or cadaveric models, engineering or technical validation studies without clinical rehabilitation outcomes, or studies that did not involve physical therapy or rehabilitation interventions were also excluded. Additionally, studies evaluating motion capture technology solely for diagnostic, surgical, ergonomic, or sports performance purposes without a rehabilitation intervention were excluded. Publications not available in English or with insufficient outcome data for extraction were also excluded.

Quality Assessment

Two independent reviewers assessed the risk of bias of the included RCTs using the Cochrane Risk of Bias 2 (RoB 2) tool [9]. Any disagreements in the risk-of-bias assessment were resolved through discussion until consensus was reached. The RoB 2 tool assesses risk of bias across five domains: the randomization process, deviations from intended interventions, missing outcome data, measurement of the outcome, and selection of the reported result. Overall risk-of-bias judgments were categorized as low risk of bias, some concerns, or high risk of bias. Among the included studies, most were judged to have a low overall risk of bias, while some studies had some concerns. Concerns were primarily related to the randomization process, deviations from intended interventions, and selection of the reported result. The domains of missing outcome data and measurement of the outcome were generally judged to have a low risk of bias across the included studies. Concerns regarding deviations from intended interventions were observed across the studies, largely because blinding of participants and therapists was not feasible due to the nature of the rehabilitation interventions. The overall risk-of-bias assessments are summarized in Table 3.

**Table 3:** Risk of bias assessment of the included randomized controlled trials using the Cochrane RoB 2 tool VR: virtual reality; RoB 2: Risk of Bias 2

Study	Randomization process	Deviations from intended interventions	Missing outcome data	Measurement of outcome	Selection of reported result	Overall risk of bias	Rationale
Shih et al. [10]	Low risk	Some concerns	Low risk	Low risk	Some concerns	Some concerns	Randomization was adequately reported, and outcome data were largely complete. However, blinding of participants and therapists was not feasible for Kinect-based exergaming, and some uncertainty remained regarding prespecification and selection of reported outcomes.
Bang and Shin [11]	Some concerns	Some concerns	Low risk	Low risk	Some concerns	Some concerns	The study provided limited detail regarding the randomization and allocation process. Blinding was unlikely to be feasible because of robot-assisted gait training. Outcome data were adequately reported, although limited information on prespecified outcomes resulted in some concerns.
Cannell et al. [12]	Low risk	Some concerns	Low risk	Low risk	Some concerns	Some concerns	The allocation process was adequately described, and outcome data were largely complete. Because participants and therapists could not be blinded to the motion-capture rehabilitation intervention, some concerns remained regarding deviations from intended interventions and selective reporting.
Liao et al. [13]	Low risk	Some concerns	Low risk	Low risk	Some concerns	Some concerns	Randomization was adequately reported, and missing outcome data were limited. Blinding of participants and therapists was not feasible for the VR intervention, while incomplete information regarding outcome selection contributed to some concerns.
Tarakci et al. [14]	Some concerns	Some concerns	Low risk	Low risk	Some concerns	Some concerns	The randomization or allocation procedure was insufficiently detailed. Blinding was not feasible for the Leap Motion rehabilitation intervention. Outcome data were generally complete, but limited reporting of prespecified outcomes resulted in some concerns.
Li et al. [15]	Low risk	Some concerns	Low risk	Low risk	Low risk	Low risk	Randomization was adequately described, and outcome data were sufficiently complete. Although participant and therapist blinding was difficult for the wearable-based intervention, outcomes were appropriately measured and reported without important concerns regarding selective reporting.
Junata et al. [16]	Low risk	Some concerns	Low risk	Low risk	Low risk	Low risk	The allocation process was adequately reported, and outcome data were complete. Blinding was not feasible for Kinect-based training; however, outcomes were appropriately assessed and reported consistently with the study objectives.
Ain et al. [17]	Low risk	Some concerns	Low risk	Low risk	Low risk	Low risk	Randomization was adequately described, and outcome data were sufficiently complete. Although participants and therapists could not be blinded to Kinect training, outcome measurement and reporting were appropriate, resulting in low overall risk.
Johnson et al. [18]	Low risk	Some concerns	Low risk	Low risk	Low risk	Low risk	The randomization process was adequately conducted, and missing data were limited. Blinding was difficult because of the rehabilitation intervention; however, biomechanical outcomes were objectively measured and appropriately reported.
Gsangaya et al. [19]	Low risk	Some concerns	Low risk	Low risk	Low risk	Low risk	Randomization was adequately reported, and outcome data were sufficiently complete. Blinding was not feasible for immersive VR rehabilitation, but outcomes were objectively assessed, and no important concerns regarding selective reporting were identified.
Gholami et al. [20]	Low risk	Some concerns	Low risk	Low risk	Low risk	Low risk	The randomization procedure was adequately reported. Participants and therapists could not reasonably be blinded to the training intervention, but outcome data were largely complete and biomechanical and functional outcomes were appropriately assessed and reported.
Guo et al. [21]	Low risk	Some concerns	low risk	Low risk	Low risk	Low risk	Randomization was adequately reported, and missing outcome data were limited. Blinding of participants and rehabilitation providers was not feasible for mobile health rehabilitation; however, functional, adherence, and quality-of-life outcomes were appropriately measured and presented.
Azab et al. [22]	Low risk	Some concerns	Low risk	Low risk	Low risk	Low risk	The randomization process was adequately reported, and outcome data were sufficiently complete. Blinding was not feasible because participants received different rehabilitation interventions, but gait and functional outcomes were appropriately assessed and reported.
Riaz et al. [23]	Low Risk	Some concerns	Low risk	Low risk	Low risk	Low risk	The allocation procedure was adequately described, and outcome data were sufficiently complete. Blinding was not feasible for the Kinect-based exercise intervention; however, balance and functional outcomes were appropriately measured and reported.
Bade et al. [24]	Low risk	Some concerns	Low risk	Low risk	Low risk	Low risk	Randomization was adequately described and outcome data were largely complete. Blinding was not feasible for movement-pattern biofeedback training, but gait biomechanical and functional outcomes were appropriately measured and reported.
Plavoukou et al. [25]	Low risk	Some concerns	Low risk	Low risk	Low risk	Low risk	The randomization process was adequately reported, and missing outcome data were limited. Blinding was not feasible for sensor-augmented telerehabilitation, but neuromuscular and functional outcomes were appropriately measured and reported.
Lim et al. [26]	Low risk	Some concerns	Low risk	Low risk	Low risk	Low risk	Randomization was adequately reported, and outcome data were sufficiently complete. Blinding of participants and therapists was not feasible for the mixed-reality exercise intervention; however, physical function, fatigue, and quality-of-life outcomes were appropriately measured and reported.

Data Synthesis

Narrative Synthesis and Statistical Analysis: Due to substantial clinical and methodological heterogeneity among the included studies, particularly in participant characteristics, intervention modalities, technologies, outcome measures, and follow-up periods, a quantitative meta-analysis was not considered appropriate. Therefore, a narrative synthesis was performed. Studies were grouped and compared according to population characteristics, intervention type and technology, outcome domains, and reported treatment effects. The findings were synthesized based on the direction and consistency of effects across studies, while differences in intervention protocols and follow-up periods were considered during interpretation. Where reported by individual studies, statistical significance, effect estimates, and confidence intervals were summarized descriptively. No pooled effect estimates were calculated.

The narrative synthesis followed a structured approach. First, the included studies were grouped according to the primary technology or technology-assisted rehabilitation approach used, including marker-based and markerless motion capture, Kinect, Leap Motion, wearable sensors and IMUs, virtual reality or mixed reality, robot-assisted systems, movement biofeedback, mobile health, and sensor-augmented telerehabilitation. Second, studies were categorized according to rehabilitation population and clinical condition, including neurological, musculoskeletal, pediatric, geriatric, and oncological rehabilitation. Third, findings were organized according to the principal outcomes assessed, including gait and spatiotemporal parameters, balance and postural control, joint kinematics, movement quality, functional mobility, upper- and lower-limb function, pain, physical activity, and quality of life.

For each outcome, the direction and consistency of reported findings were considered by comparing the intervention and comparator groups within individual studies. Findings were interpreted in relation to the methodological characteristics and risk-of-bias judgments of the included studies. Particular attention was given to whether improvements were statistically significant, whether findings were consistent across studies using similar technologies or outcomes, and whether differences in populations, intervention protocols, outcome measures, and follow-up periods could explain variation in findings. The synthesis therefore emphasized patterns and consistency of evidence rather than calculating a pooled effect estimate.

The study selection process is illustrated in the PRISMA flow diagram (Figure 3), which summarizes the identification, screening, eligibility assessment, and inclusion of studies.

**Figure 3:**
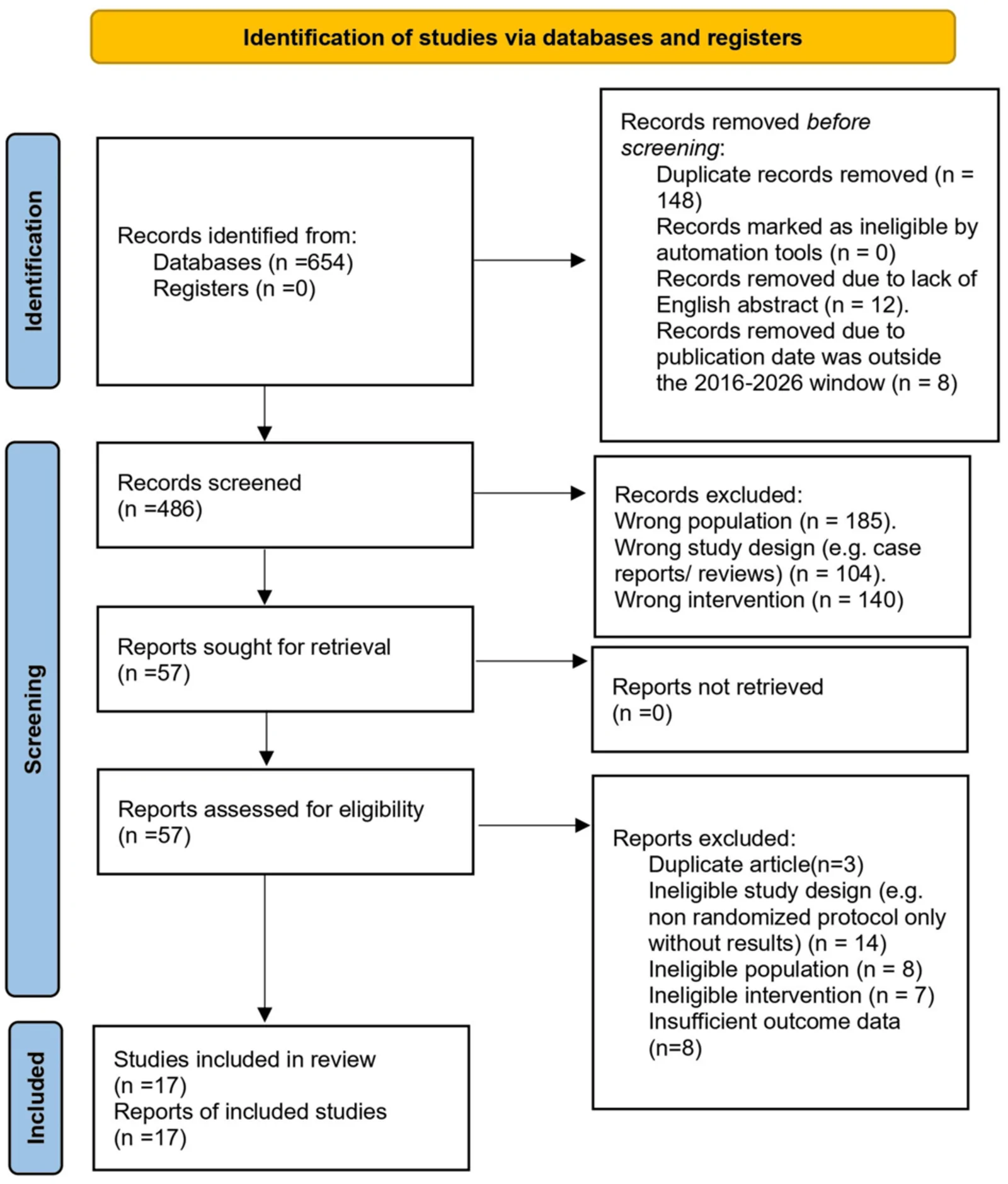
PRISMA flow diagram PRISMA: Preferred Reporting Items for Systematic Reviews and Meta-Analyses

Results

Study Selection and Characteristics

Due to substantial clinical and methodological heterogeneity among the included studies, particularly in participant characteristics, rehabilitation interventions, motion-capture technologies, outcome measures, and follow-up durations, a narrative synthesis was performed. Quantitative meta-analysis was not considered appropriate because pooling of the data could have produced misleading estimates of treatment effects.

A total of 654 records were identified through database searching. After removal of 148 duplicate records, 12 records lacking an English-language abstract, and eight records published outside the predefined 2016-2026 period, 486 records remained for screening. Following title and abstract screening, 429 records were excluded because of an ineligible population (n = 185), inappropriate study design (n = 104), or inappropriate intervention (n = 140). Fifty-seven reports were sought for retrieval, all of which were retrieved and assessed for eligibility. Forty reports were subsequently excluded because of duplicate publication (n = 3), ineligible study design (n = 14), ineligible population (n = 8), ineligible intervention (n = 7), or insufficient outcome data (n = 8). Ultimately, 17 RCTs were included in the systematic review, as presented in Figure 3.

The included studies evaluated a variety of technology-assisted rehabilitation interventions, including Microsoft Kinect-based rehabilitation, Leap Motion Controller, robot-assisted gait training, interactive motion capture systems, wearable sensor-assisted exercise programs, virtual reality, mixed reality, movement pattern biofeedback, mobile health-based rehabilitation, and sensor-augmented telerehabilitation. These interventions were compared with conventional physiotherapy, standard rehabilitation, usual care, or other conservative rehabilitation programs.

The studies included participants with Parkinson's disease, stroke, mild cognitive impairment, physical disabilities, knee osteoarthritis, anterior cruciate ligament reconstruction, hemophilic knee arthropathy, postmenopausal osteopenia, total knee arthroplasty, and breast cancer. The outcome measures commonly assessed gait biomechanics, spatiotemporal gait parameters, joint kinematics, balance, postural stability, functional mobility, upper- and lower-limb function, limb loading symmetry, neuromuscular performance, physical activity, pain, quality of life, and disease-specific functional assessment scales.

Overall, the findings suggested that integrating biomechanical analysis and motion capture technology into rehabilitation programs was associated with improvements in movement quality and functional outcomes across several rehabilitation settings. Most studies reported improvements in one or more outcomes, including gait, balance, joint kinematics, postural control, functional mobility, upper- and lower-limb motor function, physical activity, and quality of life, compared with conventional rehabilitation or other comparator interventions. Several studies also reported improvements in treatment adherence, movement symmetry, and measures related to fall or fracture risk following technology-assisted rehabilitation.

Effects of Interventions

As a quantitative meta-analysis was not performed because of clinical and methodological heterogeneity, the findings are presented as a narrative synthesis. Overall, the included studies consistently demonstrated that biomechanical analysis and motion capture technologies were effective adjuncts to conventional physical therapy across a variety of rehabilitation settings. Motion capture-based interventions improved gait biomechanics, balance, postural stability, movement quality, functional mobility, and rehabilitation performance, while also enhancing patient engagement and exercise adherence.

Despite the positive findings, variations in participant characteristics, rehabilitation protocols, motion capture technologies, intervention duration, and outcome measures should be considered when interpreting the results. These differences highlight the need for further high-quality, large-scale RCTs to establish standardized clinical guidelines and determine the long-term effectiveness of biomechanical analysis and motion capture technology in physical therapy and rehabilitation, as summarized in Table 4.

**Table 4:** Overview of the 17 included studies RCT: randomized controlled trial; VR: virtual reality; WOMAC: Western Ontario and McMaster Universities Osteoarthritis Index; ACL: anterior cruciate ligament; TKA: total knee arthroplasty

Study	Study design	Sample size	Population	Intervention	Outcome measures	Main findings
Shih et al. [10]	Single-blinded RCT	20	Individuals with Parkinson's disease	Kinect-based balance exergaming vs. conventional balance training	Berg Balance Scale [27], timed up and go [28], postural stability	Kinect training significantly improved balance, postural stability, and mobility
Bang and Shin [11]	Pilot RCT	30	Chronic stroke patients	Robot-assisted gait training vs. conventional gait training	Spatiotemporal gait parameters, Berg Balance Scale	Significant improvements in gait parameters and balance were observed in the intervention group
Cannell et al. [12]	RCT	73	Inpatient stroke patients	Interactive motion capture-based rehabilitation + conventional therapy vs. conventional therapy	Functional mobility, balance, gait, upper limb function	Motion capture rehabilitation was feasible and significantly improved functional outcomes comparable to conventional rehabilitation
Liao et al. [13]	RCT	36	Older adults with mild cognitive impairment	Virtual reality-based physical and cognitive training vs. conventional exercise	Executive function, dual-task gait performance	VR training significantly improved executive function and dual-task gait performance
Tarakci et al. [14]	RCT	32	Children and adolescents with physical disabilities	Leap motion controller-based upper limb rehabilitation vs. conventional therapy	Upper extremity functional index [29], hand function	Leap Motion training significantly improved upper extremity function and motivation
Li et al. [15]	RCT	51	Individuals with knee osteoarthritis	Wearable sensor-assisted exercise program vs. usual care	Physical activity, pain, WOMAC [30], self-management	Wearable program significantly improved physical activity and knee function
Junata et al. [16]	RCT	93	Stroke survivors	Kinect-based rapid movement training vs. conventional rehabilitation	Balance recovery, fall risk, reaction time	Significant improvement in balance recovery and reduced fall risk
Ain et al. [17]	RCT	42	Chronic stroke patients	Xbox Kinect training + conventional physiotherapy vs. conventional physiotherapy	Upper limb motor function, Fugl-Meyer assessment [31]	Kinect training significantly enhanced upper limb motor recovery
Johnson et al. [18]	RCT	44	Patients after ACL reconstruction	Functional resistance training vs. standard rehabilitation	Knee kinematics, gait symmetry, kinetics	Improved knee angle symmetry, joint moments, and gait biomechanics
Gsangaya et al. [19]	Prospective RCT	48	Patients after ACL reconstruction	Immersive virtual reality rehabilitation vs. conventional rehabilitation	Limb loading symmetry, functional outcomes	VR rehabilitation produced greater improvements in limb loading and function
Gholami et al. [20]	RCT	60	Athletes after ACL reconstruction	Differential training vs. visuomotor training	Functional performance, landing biomechanics, psychological outcomes	Both interventions improved function; differential training showed superior biomechanical outcomes
Guo et al. [21]	RCT	120	Patients after ACL reconstruction	Mobile health-based home rehabilitation vs. routine rehabilitation	Knee function, adherence, quality of life	Mobile rehabilitation significantly improved early functional recovery and treatment adherence
Azab et al. [22]	RCT	40	Adolescents with hemophilic knee arthropathy	Kinect-based virtual reality training vs. conventional physiotherapy	Gait characteristics, functional ability, physical activity	Significant improvements in gait, function, and activity levels were observed
Riaz et al. [23]	RCT	80	Postmenopausal women with osteopenia	Kinect-based virtual reality exercise vs. conventional exercise	Bone mineral density, fracture risk, balance	Kinect-based training significantly improved bone health, balance, and reduced fracture risk
Bade et al. [24]	RCT	150	Patients after total knee arthroplasty	Movement pattern biofeedback training + conventional rehabilitation vs. conventional rehabilitation	Gait biomechanics, functional mobility, pain	Biofeedback training significantly improved gait mechanics and movement quality after TKA
Plavoukou et al. [25]	RCT	60	Patients with knee osteoarthritis	Sensor-augmented telerehabilitation vs. conventional physiotherapy	Neuromuscular performance, WOMAC, psychosocial outcomes	Sensor-based telerehabilitation significantly improved neuromuscular and functional outcomes
Lim et al. [26]	RCT	60	Breast cancer survivors	Kinect-based mixed reality exercise program vs. conventional exercise	Physical function, quality of life, fatigue	Mixed reality exercise significantly improved physical function and quality of life

Assessment of Risk of Bias

Two independent reviewers assessed the risk of bias of the included RCTs using the Cochrane RoB 2 tool [9]. Any disagreements in the risk-of-bias assessment were resolved through discussion until consensus was reached.

To ensure the credibility of the synthesized data, a risk-of-bias evaluation was performed for each selected RCT. The RoB 2 tool was used to assess the methodological quality and internal validity of the included studies across five domains: the randomization process, deviations from intended interventions, missing outcome data, measurement of the outcome, and selection of the reported result.

Overall, 12 studies were judged to have a low overall risk of bias, while five studies had some concerns; none was judged to have a high risk of bias. The main concerns were related to insufficient reporting of randomization procedures, the unavoidable lack of participant and therapist blinding in technology-assisted and exercise-based rehabilitation, and limited information regarding prespecified outcome selection. Missing outcome data and outcome measurement were generally considered to have a low risk of bias. These findings indicate that the included evidence was predominantly of low methodological risk, although some methodological uncertainties should be considered when interpreting the synthesized results. Detailed study-level judgments and supporting rationales are provided in Table 3.

 Discussion

Musculoskeletal Rehabilitation

Among the included studies, substantial evidence supports the integration of biomechanical analysis and motion capture technology in musculoskeletal rehabilitation. Patients undergoing rehabilitation following anterior cruciate ligament reconstruction, total knee arthroplasty, and knee osteoarthritis demonstrated significant improvements in gait biomechanics, limb-loading symmetry, neuromuscular control, movement quality, pain, and functional performance following technology-assisted rehabilitation. Biomechanical analysis and motion capture technologies enabled objective movement assessment and, in several studies, provided real-time feedback that facilitated correction of abnormal movement patterns, thereby improving movement quality and functional performance and improving rehabilitation outcomes compared with conventional physiotherapy alone [15,17-21,31].

Neurological Rehabilitation

Motion capture technology also demonstrated considerable effectiveness in neurological rehabilitation. Studies involving individuals with stroke, Parkinson's disease, and mild cognitive impairment consistently reported improvements in gait performance, balance, postural stability, upper-limb function, reaction time, executive function, and functional mobility following Kinect-based rehabilitation, robot-assisted gait training, virtual reality, and motion capture-based interventions [11,12,14,16,23,27,28]. These findings suggest that technology-assisted rehabilitation facilitates repetitive task-specific practice, promotes neuroplasticity, and provides objective movement analysis that enhances clinical decision-making and patient recovery.

Pediatric Rehabilitation

Evidence from pediatric rehabilitation indicates that motion capture technology is both feasible and effective for improving motor function in children and adolescents with physical disabilities. Leap Motion Controller-based rehabilitation significantly enhanced upper-extremity function and hand performance, while Kinect-based virtual reality training improved gait characteristics, functional ability, and physical activity in adolescents with hemophilic knee arthropathy [13,24]. These findings support the use of interactive motion capture systems to increase patient motivation, engagement, and functional recovery in pediatric rehabilitation.

Geriatric Rehabilitation

The included studies also demonstrated promising applications of motion capture technology in geriatric rehabilitation. Virtual reality-based physical and cognitive training improved executive function and dual-task gait performance in older adults with mild cognitive impairment, whereas wearable sensor-based rehabilitation enhanced physical activity, self-management, and knee function in individuals with knee osteoarthritis [14,15]. Furthermore, Kinect-based virtual reality exercise significantly improved balance, bone mineral density, and fracture risk in postmenopausal women with osteopenia, while mixed reality exercise programs improved physical function, fatigue, and quality of life among breast cancer survivors [25,26]. Collectively, these findings indicate that technology-assisted rehabilitation may contribute to improved functional independence, mobility, and quality of life in older adults.

Overall Interpretation

Overall, the evidence included in this review suggests that biomechanical analysis and motion capture technology provide objective movement assessment, facilitate individualized rehabilitation, improve patient engagement through real-time feedback, and enhance functional outcomes across musculoskeletal, neurological, pediatric, and geriatric rehabilitation settings. Despite encouraging results, methodological heterogeneity, differences in intervention protocols, motion capture systems, outcome measures, and relatively small sample sizes in several studies limit direct comparisons. Therefore, future multicenter RCTs using standardized biomechanical outcome measures and longer follow-up periods are required to strengthen the evidence base and support routine clinical implementation of these technologies [10-31].

Clinical Implications

The findings of this systematic review suggest that biomechanical analysis and motion capture technology can enhance physical therapy by providing objective movement assessment and real-time feedback, leading to improved gait, balance, movement quality, and functional outcomes. These technologies have demonstrated clinical benefits across musculoskeletal, neurological, pediatric, and geriatric rehabilitation. However, further high-quality multicenter RCTs and standardized clinical protocols are required before their widespread implementation in routine rehabilitation practice.

Limitations

This systematic review has several limitations. The included studies exhibited considerable heterogeneity in participant characteristics, intervention protocols, motion capture technologies, outcome measures, and follow-up durations, which limited direct comparison and precluded quantitative meta-analysis. In addition, several studies had relatively small sample sizes and were conducted at single centers, which may affect the generalizability of the findings. Only English-language, peer-reviewed RCTs were included, potentially introducing language and publication bias. Furthermore, variations in biomechanical assessment methods and rehabilitation protocols across studies may have influenced the consistency of the reported outcomes.

Future Scope

Future research should focus on conducting large, multicenter RCTs with standardized biomechanical outcome measures and longer follow-up periods to strengthen the evidence for the clinical effectiveness of biomechanical analysis and motion capture technology in rehabilitation. Further studies should also evaluate the cost-effectiveness, long-term clinical outcomes, and integration of emerging technologies such as AI, markerless motion capture, wearable sensors, and tele-rehabilitation across diverse patient populations. The development of standardized clinical protocols will facilitate the wider implementation of these technologies in routine physical therapy practice.

## Conclusions

This systematic review demonstrates that the integration of biomechanical analysis and motion capture technology into physical therapy and rehabilitation has the potential to improve objective movement assessment and enhance functional outcomes across musculoskeletal, neurological, pediatric, and geriatric populations. The included RCTs consistently reported improvements in gait, balance, movement quality, postural control, and functional performance when these technologies were incorporated into rehabilitation programs. Despite these encouraging findings, variability in study methodologies, intervention protocols, and outcome measures highlights the need for standardized clinical protocols and further high-quality multicenter RCTs. Overall, biomechanical analysis and motion capture technology represent promising tools that can support evidence-based rehabilitation and contribute to more personalized and effective physical therapy practice.
